# Evaluating the Suitability of the Low Energy Availability in Females Questionnaire (LEAF-Q) for Female Football Players

**DOI:** 10.1186/s40798-023-00605-4

**Published:** 2023-07-13

**Authors:** Marcus S. Dasa, Oddgeir Friborg, Morten Kristoffersen, Gunn Pettersen, Jørn V. Sagen, Jorunn Sundgot-Borgen, Jan H. Rosenvinge

**Affiliations:** 1 Department of Health and Care Sciences, UiT – The Arctic University of Norway, Tromsø, Norway; 2 Department of Psychology, UiT – The Arctic University of Norway, Tromsø, Norway; 3 Department of Sport, Food and Natural Sciences, Western Norway University of Applied Sciences, Bergen, Norway; 4 Department of Medical Biochemistry and Pharmacology, Haukeland University Hospital, Bergen, Norway; 5 Department of Clinical Sciences, University of Bergen, Bergen, Norway; 6 Institute of Sports Medicine, Norwegian School of Sports Sciences, Oslo, Norway

**Keywords:** Low energy availability, Football, Soccer, Female, Relative energy deficiency in sports, Female athlete triad, Athlete health

## Abstract

**Background:**

The Low Energy Availability in Females Questionnaire (LEAF-Q) is a screening tool developed to detect endurance athletes and dancers at risk for development of persistent low energy availability (LEA) and the female athlete triad (Triad). This study investigated the applicability of the LEAF-Q in a cohort of sixty professional female football players.

**Methods:**

The participants were classified as *at risk* (≥ 8) or *not at risk* (< 8) for persistent LEA and the Triad according to their LEAF-Q score, before being compared. Receiver operating curves were then conducted to examine the ability of the overall LEAF-Q and subcategories to correctly determine the presence of clinically defined markers of the Triad. Additionally, Youden’s index was calculated to determine the best fitting cut-off values.

**Results:**

Thirty-two percent of participants were classified as *at risk* by the LEAF-Q. We found no statistically significant differences between the two groups for any markers associated with persistent LEA. Except for acceptable accuracy in determining menstrual status, all other LEAF-Q components exhibited poor accuracy and predictive values. Youden’s index scores imply that increasing the overall and injury cut-off values to ≥ 10 and ≥ 5 respectively, would yield increased performance.

**Conclusions:**

Our findings do not support the use of the LEAF-Q for the purpose of detecting LEA and Triad conditions among female football players.

## Key points

- The LEAF-Q is a screening tool developed to identify female endurance athletes and dancers at risk for development of low energy availability and the female athlete triad. The questionnaire has been used in a wide range of athlete populations, including female football players, without sufficient knowledge about its applicability.
- The prevalence of acute and impact injuries in football, compared to endurance sports and dancing, may introduce substantial bias, leading to artificially inflated scores on the questionnaire.
- Our findings do not support the use of the LEAF-Q for the purpose of detecting symptoms of the female athlete triad and LEA among female football players.

## Introduction

Energy availability (EA) is defined as the difference between energy intake (EI) and exercise energy expenditure. It is expressed relative to an individual’s fat-free mass (FFM) and represents the residual amount of energy available to sustain all physiological functions [1]. Consequently, low energy availability (LEA) denotes the state where the body receives insufficient energy to optimally perform these functions. Despite emerging evidence indicating individual variation in response to LEA, it is commonly defined as < 30 kcal/kg^−1^ FFM/day^−1^ [2]. Persistent or severe LEA are recognized as the etiological underpinning of both the female athlete triad (Triad) and Relative Energy Deficiency in Sport (REDs) [3, 4]. The Triad encompasses three interrelated conditions: EA, menstrual function and bone health, all ranging on a continuum from health to disease, with LEA as a causal factor of Triad dysfunction [5]. The REDs model, however, describes a broader range of potential consequences affecting both health and sports performance among males and females, caused by LEA [3]. Despite discussions regarding the scientific rigor and causal evidence supporting the REDs model [6], it now contributes to the International Olympic Committee’s (IOC) consensus statement and guidelines for supporting athletes’ health [3].

The Low Energy Availability in Females Questionnaire (LEAF-Q) is a screening tool developed for endurance athletes and dancers, and is validated against clinical markers of the Triad and persistent LEA [7]. The LEAF-Q consists of 25 items that assesses three subcategories: injuries, gastrointestinal symptoms, and reproductive/menstrual function, respectively. The original study recommended that additional validation of the questionnaire is necessary before utilizing it beyond the intended population [7]. The clinical utility of the LEAF-Q was recently examined in a mixed-sport cohort of athletes within individual sports, as well as netball and water polo [8]. It was concluded that the LEAF-Q was suitable to “rule out” LEA-related conditions in athletes who scored below the originally published cut-off value, however, it failed to identify athletes “at risk” of the Triad or LEA with its associated symptoms.

Football is characterized by a mixture of high- and low-intensity efforts and actions, and is played on a relatively large pitch (90–120 m length, 45–90 m width) [9]. High-level female players usually cover between 9–11 km during a game [10]. As such, football sets itself apart from the majority of contemporary individual and team sports. Although LEA and its associated symptoms are thought to be most prevalent in endurance and weight-sensitive sports [3], studies have reported a wide range of prevalence estimates among female footballers, depending on the measurement methods applied [11–16]. This includes studies employing the LEAF-Q to screen for the Triad and LEA, despite insufficient evidence for its application in this population [12, 13, 17]. As LEA and subsequently REDs may have profound ramifications for athletes’ health and performance, there is a need to investigate the suitability of commonly used measurement instruments among female football players. Therefore, the purpose of this study was to evaluate the applicability of the LEAF-Q as a screening tool for female football players. Using a cohort of professional female football players, we examined the capacity of the LEAF-Q to identify markers associated with the Triad and persistent LEA and to correctly classify players *at risk* for these conditions.

## Methods

### Study Design

In the present cross-sectional study, we conducted a comprehensive analysis of multiple clinical markers related to the Triad [7] and previously published literature on the subject [18–21]. The data collection was conducted between October 2021 and May 2022. Within two weeks each participant completed all measurements across two subsequent days.

### Participants

Sixty female football players from three Norwegian teams were included in the study. Eight players were currently representing the Norwegian senior national team, while another eight players represented their designated youth national team. The participants were classified as tier 3 (national level) or 4 (international level) according to the athlete classification framework [22].

### Body Composition and BMD

Body composition (% fat mass, FFM) and bone mineral density (BMD) were assessed in a fasting state using Dual-Energy X-Ray Absorptiometry (DXA; Prodigy, Encore, SP 4.1, version 18, GE medical systems, Madison, Wisconsin, USA), according to best practice guidelines [23]. Before completing the scan, body weight (Seca 869, Hamburg, Germany) ± 0.1 kg and height (Seca, Hamburg, Germany) was recorded. Participants, dressed in minimal attire (i.e., tights and t-shirt), were situated in the supine position, ensuring their body was properly aligned with the central longitudinal axis of the scan table. Both arms were positioned alongside the body, in neutral position to minimize overlapping of anatomical structures. Participants first underwent a total body scan for assessment of body composition. This was followed by an anteroposterior scan of the lumbar spine (L1-4) and bilateral hip densitometry to evaluate BMD. Automatic analysis was performed using the manufacturer’s software (Encore, SP 4.1) and manually adjusted if indicated. All measurements and analyses were conducted by the same certified technician to avoid inter-rater variability and error.

### Energy Availability

Measures of EA were estimated through resting metabolic rate (RMR). Specifically, RMR < 30 kcal/kg^−1^ FFM/day^−1^ or RMR_Ratio_ < 0.90 using the measured value, and the Cunningham equation was considered indicative of LEA [24, 25]. RMR is considered a viable option for estimating EA, when used in combination with other markers, as well as being strongly correlated with energy deficiency and amenorrhea in exercising women [26, 27]. Due to significant challenges associated with the direct measurement of EA, and the lack of a gold standard method, it was deemed appropriate to utilize this surrogate marker [28, 29].

RMR was measured with the participants arriving at the test facility by motorized transportation between 06 and 09 a.m. in an overnight fasted state. Participants were placed in a silent room, in the supine position for 5 min, before a ventilated canopy hoodie (Vyntus CPX, CareFusion, Hoechberg, Germany, Sentrysuit v. 2.21.4) was positioned. Oxygen consumption (VO_2_) and carbon dioxide production (VCO_2_) were then measured for 25 min, where the average value for the last 20 min was used to assess RMR.

### Screening Instruments

Participants filled out the LEAF-Q after completion of RMR and DXA measurement, at the testing facility. The questionnaire was administered with a portable tablet, using a digital encrypted platform (Nettskjema, University of Oslo, Norway). Participants were classified as *at risk* (total score ≥ 8) or *not at risk* (< 8) for the Triad, according to the LEAF-Q scoring system [7]. Further, in accordance with the original publication, the LEAF-Q cut-off values associated with increased risk for Triad dysfunction were applied in the same manner (Injuries(≥ 2), gastrointestinal symptoms (≥ 2) and menstrual function (≥ 4)). As LEA may be present with or without disordered eating [30], the participants also completed the Eating Disorder Examination Questionnaire (EDE-Q 11). This has been extensively used to assess self-reported eating behavior pathology [31]. Information on the history of stress fractures, which has been strongly linked with LEA [32], was obtained through a custom-made question that specifically inquired about the injury position and frequency.

### Menstrual Function

The LEAF-Q was used to determine menstrual status, i.e., eumenorrheic or amenorrheic (oligomenorrhea was considered as amenorrhea). Menstrual status could not be determined in participants who reported usage of hormonal contraception (55%). As it was not considered ethically acceptable to request cessation of hormonal contraceptive usage, the menstrual status was only classified in 27 of the participants (45%).

### Blood Samples

After an overnight fasting period (8–10 h), blood was collected for both plasma and serum samples [33]. These samples were stored in Biobank Haukeland, Laboratory Medicine and Pathology, Haukeland University Hospital, Bergen, Norway, in 3.5 ml serum/gel vacutainers before analyses. Analyses that have either been directly linked to or associated with LEA were assessed. This included glucose, insulin, thyroid stimulating hormone (TSH), free triiodothyronine (T_3_), free thyroxine (T_4_), insulin-like growth factor 1 (IGF-1), and leptin, which were analyzed at the Department of Medical Biochemistry and Pharmacology, Haukeland University Hospital, Bergen, Norway. The laboratory is accredited in compliance with ISO 15189:2012. Glucose was analyzed using Cobas 8000, TSH, free T_3_ and free T_4_ were measured with Cobas e801. Insulin and IGF-1 were analyzed using Immulite 2000 XPi, whereas leptin was determined using an enzyme-linked immunosorbent assay kit (Mediagnost Cat#E07, RRID: AB_2813737)(non-accredited analysis).

### Statistical Analyses

The statistical analyses were conducted using SPSS 28 (IBM, Armonk, NY, USA). Variables being non-normally distributed according to the Shapiro-Wilks test were described using median and range and between group variables examined with nonparametric tests (Mann–Whitney U). Otherwise, parametric tests (Welch’s test for unequal sample size) and mean ± standard deviation (SD) were reported.

Descriptive statistics are provided for the whole sample, as well as separately for participants classified as *at risk* versus *not at risk* for symptoms of the Triad, as defined by their LEAF-Q scores. The alpha level was set to < 0.05.

We used receiver operating curve (ROC) analyses to examine the ability of the LEAF-Q to correctly determine the presence of clinically defined markers of the Triad. For this purpose, we report the area under curve (AUC), sensitivity, specificity, positive predictive value (PPV) and negative predictive value (NPV), as well as the highest Youden’s index [33] locating the best cut-off value for the overall and the subcategory LEAF-Q scores. These sample-derived discriminatory properties and cut-off scores were compared to the original overall and subcategory cut-off scores, as published by the original authors [7]. The AUC estimates the overall capacity of the LEAF-Q to correctly discriminate Triad from non-Triad cases. The AUC value ranges between 0 and 1, with higher values indicating better discrimination. A non-discriminatory test has an AUC of 0.5 (50%), while higher AUC values represent better than random classification with AUC = 1.0 (100%) being perfect. AUC values > 0.70 (70%) are considered as fair, > 0.80 (80%) as good, and above > 0.90 (90%) as excellent. AUC values below 0.5 indicate reciprocal discrimination that is opposite of expected [34].

In the event of significant AUC values, precision recall curves (PRC) were additionally calculated as ROC analyses may be misleading in case of severely imbalanced data sets (e.g., skewed numbers of positive or negative cases) [35]. Since the majority of athletes generally are expected to be non-symptomatic, we were also interested in the ability of the LEAF-Q score to positively predict individuals with markers of LEA. As such, the PRC may provide additional information regarding the questionnaire’s tenability through measures of *precision* (identical to PPV in ROC) = $\frac{{{\text{true}}\;{\text{positives}}}}{{\left( {{\text{true}}\;{\text{positives}} + {\text{false}}\;{\text{positives}}} \right)}}$ and *recall* (identical to sensitivity in ROC) = $\frac{{{\text{true}}\;{\text{positives}}}}{{{\text{true}}\;{\text{positives + false}}\;{\text{negatives}}}}$. As none of these formulas involve the number of “true negative” (TN) cases, which is expected to constitute most cases in the present sample, the PRC curves are likely less biased due to the extreme skewness the true negative cases represent in the ROC curve.

Two participants were excluded from the respective analyses; one who was not able to provide blood samples (did not meet for the scheduled appointment), and one who could not provide a measure of RMR due to illness.

## Results

There were no statistically significant differences between the two groups for any of the clinical markers associated with LEA, however, free T_4_ had a P-value of 0.05. Descriptive statistics of the sample, which separates the women classified as *at risk* (LEAF-Q ≥ 8) and *not at risk* (LEAF-Q < 8) is presented in Table 1.

**Table 1:** Descriptive data for all participants and stratified into groups (at risk/low risk of LEA) based on LEAF-Q score. Brackets indicate number of participants in the different groups

Measure	Total (60)	At risk (LEAF-Q ≥ 8) (19)	Low risk (Leaf-Q < 8) (41)	*P*
Age (Years)	22.5 [16, 32]	21.0 [17, 32]	22.0 ± [16, 32]	0.69
Weight (kg)	64.1 ± 6.3	64.3 ± 5.2	64 ± 6.7	0.88
Height (cm)	168.9 ± 6.0	169.1 ± 6.3	168.8 ± 5.8	0.86
Fat mass (%)	24.4 [16, 37]	25 [18, 33]	23 [16, 37]	0.39
BMI (kg/m^2^)	22.4 [19, 29]	22.5 [19.6, 25.6]	22.4 [19.0, 29.1]	0.69
Weekly training volume (h) ^a^	12.5 ± 3.2	12.5 ± 3.2	12.7 ± 3.1	0.82
RMR (kcal)	1464 ± 225	1460 ± 237	1467 ± 224	0.91
RMR_Ratio_	0.96 ± 0.12	0.96 ± 0.15	0.95 ± 0.10	0.65
BMD lumbar spine (Z-score)	1.2 ± 1	1.0 ± 0.9	1.3 ± 1.0	0.45
BMD hip (Z-score)	2.1 ± 0.1	1.9 ± 0.8	2.1 ± 1.0	0.49
EDE-Q-11	0.8 [0, 3.8]	1 [0. 3.8]	0.6 [0, 3.4]	0.13
Leptin (ug/L)	6.5 [2.6, 32.4]	6.3 [2.7, 32.4]	6.7 [2.7,32.3]	0.83
Free T_3_ (pmol/L)	4.9 ± 0.7	4.8 ± 0.8	5.1 ± 0.6	0.24
Free T_4_ (pmol/L)	15.8 ± 1.9	15.1 ± 1.8	16.2 ± 1.8	0.05
TSH (mlU/L)	1.6 [2.8, 6.8]	1.6 [0.6, 5.0]	1.6 [0.6, 4.3]	0.98
Glucose (mmol/L)	4.6 [2.8, 6.8]	4.7 [3.8, 5.0]	4.5 ± [2.8, 6.8]	0.65
Insulin (mlU/L)	8.8 ± 8.7	7.5 ± 6.8	9.4 ± 9.5	0.45
IGF-1 (nmol(L)	29.1 ± 7.6	29.6 ± 7.2	28.9 ± 7.9	0.75

a = self-reported training volume excluding matches

BMI = body mass index, RMR = resting metabolic rate, EDE-Q = eating disorder examination questionnaire, T3 = triiodothyronine 3, T4 = thyroxine, TSH = thyroid-stimulating hormone, IGF-1 = insulin-like growth factor 1

The overall LEAF-Q score had a mean value of 7.0 ± 3.0, whereas the mean values for the subcategories were 3 ± 2.3 (injury), 2 ± 1.7 (gastrointestinal symptoms) and 2 ± 2.4 (menstrual symptoms), respectively. Moreover, 32% were classified as *at risk* for the triad (LEAF-Q total ≥ 8). For the subcategories, 68%, 55% and 15% scored above the LEAF-Q cut-off score which are associated with LEA in the original publication [6] for injury (≥ 2), gastrointestinal symptoms (≥ 2) and menstrual symptoms (≥ 4), respectively.

For the overall LEAF-Q, the AUC index was poor for the clinical markers RMR_Ratio_, BMD_lumbar_, BMD_hip_ and RMR < 30 kcal/kg^−1^ FFM/day^−1^ (AUC = 0.44 – 0.53), whereas detection of amenorrhea had a good AUC = 0.86. Table 2 provides estimates of sensitivity, specificity, PPV and NPV, respectively. The PRC for amenorrhea showed a precision of 67% and a recall of 75%, indicating a reduction in actual precision compared to the ROC analysis in terms of identifying athletes at risk. Furthermore, the Youden’s index implies that a cut-off score ≥ 10 as opposed to ≥ 8 would be more appropriate for this cohort.

**Table 2:** Diagnostic performance of the LEAF-Q overall score to identify individuals with clinical indicators of the Triad

Measure	AUC [95% CI]	Sensitivity (%)	Specificity (%)	PPV (%)	NPV (%)
RMR_Ratio_ (< 0.90)	0.46 [0.31, 0.62]	24	63	26	60
RMR_Ratio_ (< 0.90)^a^		14	90	50	66
BMD (Z-score < − 1)					
Hip^b^	–	–	–	–	–
Lumbar spine	0.53 [0.37, 0.68]	0	68	0	98
Lumbar spine^a^		100	44	3	100
Hip	0.44 [0.28, 0.61]	0	10	0	5
Hip^a^		45	100	100	44
Lumbar spine	0.65 [0.40, 0.91]	40	69	11	93
Lumbar spine^a^		40	95	40	95
RMR < 30 kcal/kg^−1^ FFM/day^−1^	0.44 [0.29, 0.59]	28	66	37	56
RMR < 30 kcal/kg^−1^ FFM.day^−1 a^		12	91	50	59
Amenorrhea [27]	0.86 [0.69, 1.03]	75	79	60	88
Amenorrhea^a^		75	95	86	90

Cut-off values are based on consensus values derived in the literature

a = Best performing cut-off value based on Youden’s index, b = indicates that no participant presented with the condition variable, not allowing for statistical analysis

EA = energy availability, LEA = low energy availability, AUC = area under the curve, CI = confidence intervals, RMR = resting metabolic rate, FFM = fat free mass, PPV = positive predictive value, NPV = negative predictive value, BMD = bone mass density

For the subcategories, the AUC index performed poorly in detecting clinical markers of LEA for BMD_hip_, EA, and stress fracture (AUC = 0.43–0.47). AUC to detect amenorrhea was excellent (0.93), but fair (0.78) in detecting BMD_lumbar_, indicating overall good performance for these subcategories (Table 3). The PRC for amenorrhea showed a precision of 70% and a recall of 88%, while BMD_lumbar_ had a precision of 2% and recall of 100%. The Youden’s index implies that an increase in cut-off score to ≥ 5 as opposed to ≥ 4 yield a better accuracy for the injury subcategory.

**Table 3:** Diagnostic performance of the LEAF-Q subcategories (injury, gastrointestinal symptoms, and menstrual function) to identify individuals with the associated indicators of the Triad, based on the original publication

Cut off-score	AUC [95% CI]	Sensitivity (%)	Specificity (%)	PPV (%)	NPV (%)
Injury ≥ 2					
BMD (Z score < -1)					
Lumbar spine	0.78 [0.61, 0.96]	100	32	2	100
Lumbar spine^a^		100	66	5	100
Hip		–	–	–	–
History of Stress fracture	0.43 [0.25, 0.62]	54	28	18	68
BMD (Z score < 0)					
Lumbar spine	0.77 [0.59, 0.95]	80	33	10	95
Lumbar spine^a^		80	69	19	97
Hip	0.47 [0.01, 0.92]	50	31	2	95
Hip^a^		50	66	5	97
Gastrointestinal symptoms ≥ 2					
RMR < 30 kcal/kg/FFM^b^	0.45 [0.31, 0.60]	56	46	42	59
Menstrual function ≥ 4					
Amenorrhea (27)^b^	0.93 [0.83, 1.03]	88	90	78	94

*LEA* Low energy availability

a = best performing cut-off score based on Youden’s index; b = indicates that the original cut-off score is the best performing based on Youden’s index

## Discussion

The current study aimed to examine the applicability of the LEAF-Q to identify markers associated with the Triad and persistent LEA and correctly identify players *at risk* for these conditions. In terms of broad indicators associated with LEA, no statistically significant differences were observed between the groups. While T_4_, an important metabolic regulator, approached significance, this tendency was not resembled by other hormones, such as T_3_. It is notable that T_3_, more closely associated with LEA in the literature, is derived from T_4_, yet did not follow the same trend [36, 37]. It is possible that further reduction in T_4_ levels could induce changes in T_3_ within the *at risk* group, thereby exacerbating their risk for metabolic alterations associated with LEA. Melin et al. found significant differences between groups for several LEA related hormones, including leptin, T_3_ and glucose [7]. Consequently, our findings align more closely with those of Rogers et al. who found minimal variation in LEA indicators between mixed sport athletes, categorized by the LEAF-Q [8]. Pertaining to our results, the observed uniformity in LEA indicators across the groups is consistent with the general performance of the LEAF-Q in this study.

The overall performance of the LEAF-Q in detecting menstrual dysfunction was commendable, evident by an AUC of 0.86, suggesting that the original cut-off value of ≥ 8 is appropriate for this indicator. However, the questionnaires effectiveness in identifying players presenting with clinical symptoms of the Triad was suboptimal, rarely performing better than guessing by random. Further, among the recalculated cut-off scores, only amenorrhea boasted a Youdens’s index above 50%, demonstrating poor performance for the recalculated cut-off scores as well. This underscores the apparent disconnect between the perceived risk, as determined by the LEAF-Q assessment, and the tangible manifestation of Triad and LEA indicators. It is important to note that the prevalence of positive indicators for Triad and LEA was relatively low in this cohort, which is consistent with previous assumptions [38]. This could partly explain the poor diagnostic performance relative to previous investigations [7, 8]. Despite this, the LEAF-Q was still unable to accurately identify individuals without signs of LEA, strengthening the overall weak performance observed.

For the injury subcategory, the LEAF-Q demonstrated a fair AUC, as well as excellent sensitivity. Nevertheless, the specificity and PPV was very poor, showing that the LEAF-Q would fail to identify individuals with compromised markers, given a higher prevalence of the condition. The mean injury score was 3.0 ± 2.3 in the context of an overall mean score of 7.0 ± 3.8, resulting in a 68% prevalence above the ≥ 2 cut-off value. This indicates a systematic bias toward elevated injury scores among the participants. It is important to note that the LEAF-Q was originally validated for endurance athletes and dancers [7], who primarily experience overuse injuries [39, 40]. Football, on the other hand, is a high-impact sport with potential for both acute and overuse injuries [41]. This crucial distinction is not accounted for by the LEAF-Q, consequently leading to skewed scores and biased results. Furthermore, the gastrointestinal subcategory performance in detecting athletes with Reduced RMR_Ratio_ or RMR < 30 kcal/kg^−1^ FFM/day^−1^ (LEA) was very poor, with an AUC of 0.45. The original LEAF-Q included gastrointestinal symptoms as this have been reported in female athletes suffering from disordered eating and or eating disorders [42]. It is possible that these disorders are underrepresented in football, thus making gastrointestinal symptoms inappropriate as a clinical marker among female footballers.

Although the prevalence of LEA and REDs is equivocal in female football players, access to a quality screening tool is necessary. A recent investigation revealed that English female football players exhibit insufficient nutritional knowledge and express apprehension regarding carbohydrate consumption [43]. Moreover, the available literature indicates that female football players may not ingest adequate energy amounts to support optimal performance and recovery [11, 15]. Nonetheless, the existing version of the LEAF-Q lacks the necessary predictive capacity for usage among female footballers. The questionnaire was also developed before recent advancements related to REDs, primarily focusing on the causal relationship between LEA, menstrual disorders, and BMD [4, 20]. As such, resources should be allocated to further exploration of reliable surrogate markers in line with future developments. Connected to this, a recent debate has also emerged about the BMD Z-score thresholds for high-impact sports like football. As athletes experiencing high amounts of mechanical loading are expected to have elevated BMD compared to controls, utilizing the same threshold of < -1 might mask potential consequences of persistent LEA in football players [44]. Nevertheless, increasing the BMD threshold to Z-score < 0 did not significantly change or increase the prevalence estimates in our cohort.

A number of participants in the present study were unable to provide direct assessment of EA. Currently, there is no recognized gold standard method to quantify EA and there are significant constraints associated with direct measurement, particularly in intermittent sports [28, 45]. Hence, we decided to apply a surrogate marker to quantify EA, diverging from the approach of the original study. RMR may be prone to confounding factors such as energy status and recent training intensity/volume [46]. Together with assumptions related to RMR_Ratio_, this could potentially affect our results. As a cross-sectional study, outcome variables will reflect the training and match load at the time of testing. The study included several teams, which were tested at different periods during the year (October-May). Limits of the cross-sectional design may therefore, to some extent, be counterbalanced by catching variability of physiological load across seasons and teams. Lastly, due to contraceptive usage among the participants, information about menstrual irregularities could not be attained by all. This raises the risk of underestimating the prevalence of actual amenorrhea in the cohort. This is, however, reflective of the situation in real world settings [47, 48].

## Conclusion

In a diverse array of athletic cohorts, the utilization of the LEAF-Q screening tool persists, despite the fact that its validation remains restricted to endurance athletes and dancers. The poor predictive power of the LEAF-Q does not support its use for the purpose of detecting symptoms of the Triad and LEA with its associated symptoms among female football players. Consequently, the present study may serve to reconsider the interpretation of previous findings where the LEAF-Q has been used to estimate the prevalence of the Triad and LEA in populations for which it has not been validated. Future development of health screening tools for football players should consider the impact and injury mechanisms, as compared to non-contact sports.

## Data Availability

Any data requests can be directed to the corresponding author upon reasonable request.

## Abbreviations

EA: Energy availability
EI: Energy intake
FFM: Fat free mass
LEA: Low energy availability
REDs: Relative energy deficiency in sport
TRIAD: The female athlete triad
LEAF-Q: Low energy availability in female questionnaire
BMD: Bone mass density
DXA: Dual-energy X-ray absorptiometry
RMR: Resting metabolic rate
T_3_: Triiodothyronine
T_4_: Thyroxine
ROC: Receiver operator curve
AUC: Area under the curve
PPV: Positive predictive value
NPV: Negative predictive value
PRC: Precision recall curve
TSH: Thyroid stimulating hormone
IGF-1: Insulin-like growth factor 1
EDE-Q: Eating disorder examination questionnaire
BMI: Body mass index
